# Detecting COVID-19 Clusters at High Spatiotemporal Resolution, New York City, New York, USA, June–July 2020

**DOI:** 10.3201/eid2705.203583

**Published:** 2021-05

**Authors:** Sharon K. Greene, Eric R. Peterson, Dominique Balan, Lucretia Jones, Gretchen M. Culp, Annie D. Fine, Martin Kulldorff

**Affiliations:** New York City Department of Health and Mental Hygiene, Long Island City, New York, USA (S.K. Greene, E.R. Peterson, D. Balan, L. Jones, G.M. Culp, A.D. Fine);; Harvard Medical School, Boston, Massachusetts, USA (M. Kulldorff)

**Keywords:** 2019 novel coronavirus disease, coronavirus disease, COVID-19, severe acute respiratory syndrome coronavirus 2, SARS-CoV-2, viruses, respiratory infections, zoonoses, community outreach, contact tracing, disease outbreaks, epidemiology, geographic mapping, New York City, New York, USA, public health surveillance, space-time clustering

## Abstract

A surveillance system that uses census tract resolution and the SaTScan prospective space-time scan statistic detected clusters of increasing severe acute respiratory syndrome coronavirus 2 test percent positivity in New York City, NY, USA. Clusters included one in which patients attended the same social gathering and another that led to targeted testing and outreach.

Spatiotemporal analysis of high-resolution coronavirus disease (COVID-19) data can help health officials monitor disease spread and target interventions (*1*,*2*). Publicly available data have been used to detect COVID-19 spatiotemporal clusters at county and daily resolution levels across the United States (*3*; R. Amin et al., unpub. data, https://doi.org/10.1101/2020.05.22.20110155) and spatial clusters at ZIP code resolution in New York City (NYC), New York, USA (*4*).

For routine surveillance, the NYC Department of Health and Mental Hygiene (DOHMH) uses the case-only space-time permutation scan statistic (*5*) in SaTScan (https://www.satscan.org) to detect new outbreaks in the context of minimal or stable citywide incidence of reportable diseases (*6*) (e.g., Legionnaires’ disease [*7*] and salmonellosis [*8*]). Given wide testing variability, case-only analyses could be poorly suited for COVID-19 monitoring because true differences in disease rates across space and time would be indistinguishable from changing testing rates. We sought to detect in near real-time—regardless whether overall transmission was increasing, decreasing, or steady—newly emerging or re-emerging hotspots (i.e., areas where COVID-19 diagnoses, adjusted for the number of persons tested, were increasing or not decreasing as quickly relative to elsewhere in the city).

## The Study

Clinical and commercial laboratories are required to report all severe acute respiratory syndrome coronavirus 2 (SARS-CoV-2) molecular test results (positive, negative, indeterminate) for New York state residents to the New York State Electronic Clinical Laboratory Reporting System (*9*). For NYC residents, this reporting system transmits reports to DOHMH. Laboratory reports include specimen collection date and patient demographics, including residential address, which we geocoded by census tract. Patient symptoms and illness onset date, if any, are obtained through interviews, although not all patients are interviewed.

To detect emerging clusters, the space-time scan statistic uses a cylinder in which the circular base covers a geographic area and the height corresponds to time (*10*). This cylinder is moved, or scanned, over space and time to cover different areas and periods. At each position, the number of cases inside the cylinder is compared with the expected count under the null hypothesis of no clusters by using a likelihood function, and the position with the maximum likelihood is the primary candidate for a cluster. The statistical significance of this cluster is then evaluated, adjusting for the multiple testing inherent in the many cylinder positions evaluated.

To quickly detect emerging hotspots, prospective analyses are conducted daily (*11*). To adjust for the multiple testing stemming from daily analyses, recurrence intervals are used instead of p values (*12*). A recurrence interval of D days means that under the null hypothesis, if we conduct the analysis repeatedly over D days, then the expected number of clusters of the same or larger magnitude is 1.

The space-time scan statistic can be used with different probability models; we used the Poisson model (*10*), adjusting not for population size (which would fail to account for changing testing rates) but rather for persons tested. Because the goal was to detect newly emerging hotspots rather than areas with consistently high percent positivity, we further adjusted analyses nonparametrically for purely geographic variations that were consistent over time. Fitting a log-linear function, we also adjusted for citywide temporal trends in percent positivity because the goal was to detect local hotspots rather than general citywide trends. For each day and location, the expected count was calculated as the number of persons tested × temporal trend function × a location-specific constant to ensure that, summed over all days in the study period, the location has the same number of observed and expected cases. To prioritize quickly emerging clusters to identify epidemiologic linkages, we used a short maximum temporal window of 7 days. To detect sustained clusters to inform place-based resource allocation, starting July 15, we also ran secondary analyses with a maximum temporal window of 21 days.

We developed SAS code (SAS Institute, https://www.sas.com; https://github.com/CityOfNewYork/communicable-disease-surveillance-nycdohmh) to generate daily input and parameter files (Table 1; Appendix Table). The SAS code then invoked SaTScan in batch mode, read analysis results back into SAS for further processing, output files to secured folders (including patient line lists with demographics and map and time-trend visualizations), and sent an investigator notification email.

**Table 1:** Input file specifications for SARS-CoV-2 test percent positivity cluster detection analyses in New York City, NY, USA, June–July 2020*

Feature	Selection	Notes
Geographic aggregation	Census tract (defined by using US Census 2010 boundaries) of residential address at time of report	With less aggregated data, the more precisely areas with elevated rates can be identified. New York City has 2,165 census tracts located on land. If geocoding is not feasible, then ZIP code could be used but with a loss of spatial precision.
Case file	Unique persons reported with a positive result for a molecular amplification detection (PCR) test for SARS-CoV-2 RNA in a clinical specimen. Retain specimen collection date of first positive test.	Confirmed COVID-19 cases (https://cdn.ymaws.com/www.cste.org/resource/resmgr/2020ps/ Interim-20-ID-01_COVID-19.pdf)
Population file	Unique persons reported with a molecular amplification detection (PCR) test for SARS-CoV-2 RNA in a clinical specimen. For persons who ever tested positive, retain specimen collection date of first positive test. Otherwise, retain most recent specimen collection date. For a given census tract and date, if no specimens were collected, then include in file as having 0 population.	Necessary to control for spatial and temporal variability in testing access. A census-based population denominator would not control for variable testing uptake because the number of persons tested is not necessarily proportional to population size.
Omissions from input files	Residents of long-term care facilities, correctional facilities, facilities housing people with developmental disabilities, or homeless shelters; persons whose home address matches selected providers or facilities; persons diagnosed in the 14 d before a more recent case residing in the same building identification number from geocoding; persons with COVID-19 illness onset (where available from patient interview) >14 d before specimen collection.	To focus on detecting recent community-based transmission, exclude residents of congregate settings because building-level clusters are detected by using other methods (*13*), persons whose listed home address is not a residence, >1 case/building, and patients whose diagnosis was made long after illness onset.
Date of interest for analysis	Specimen collection date	Defining reportable disease clusters according to when patients became ill is preferred, although a large proportion of COVID-19 infections are asymptomatic. Specimen collection date is the earliest date available for the study population of persons tested.
Study period	21 d for analysis to support prioritization of case investigations; since June 1, 2020, for analysis to support place-based resource allocation	Defining a study period >3 times the maximum temporal window helps with statistical power. Extending the study period further may decrease the accuracy of the log-linear temporal trend adjustment but might be of interest for detecting more prolonged clusters. If citywide percent positivity reaches an inflection point (e.g., begins to increase again after a period of decrease), the study period would need to be either temporarily shortened and reset after that inflection point to preserve suitability of a log-linear temporal trend adjustment or a nonparametric temporal trend adjustment could be used. For a longer temporal window, June 1, 2020, was selected as the earliest date when citywide percent positivity trend seemed stable without an inflection point. After 63 d elapsed from June 1, 2020, switched to 63-d rolling study period until next inflection point was reached.
Lag for data accrual	3 d	Given lags between specimen collection and report, exclude very incomplete data at end of study period when estimating the temporal trend. Three days is the minimum lag possible to preserve a timely analysis while allowing for at least some data to be reported, geocoded, and analyzed before open of business.

*The prospective Poisson-based space-time scan statistic was used**. **COVID-19, coronavirus disease; SARS-CoV-2, severe acute respiratory syndrome coronavirus 2.

We launched the system on June 11, 2020, and 2 clusters detected by July 31 prompted public health action (Table 2). First, on June 22, in the context of waning case counts citywide, the only cluster detected was of 6 patients residing in a 0.6-km radius, all with specimens collected on June 17 (Figure, panel A). Consequently, DOHMH staff interviewed patients to collect and compare potential common exposures, such as attending the same event or visiting the same location. On June 23, a DOHMH surveillance investigator (D.B.) determined that 2 patients had attended the same gathering, where recommended social distancing practices had not been observed. In response, DOHMH launched an effort to limit further transmission, including testing, contact tracing, community engagement, and health education emphasizing the importance of isolation and quarantine. No other epidemiologic linkages were identified after attempts to investigate ≈65 additional clusters detected through July 2020. Second, detection on July 15, of a sustained cluster (lasting >1 week) with a high percent positivity (Figure, panel B) contributed to geographically targeted testing, outreach, and education, as part of NYC’s hyper-local plan to prevent COVID-19 transmission (*14*).

**Table 2:** Spatiotemporal clusters of SARS-CoV-2 test percent positivity prospectively detected and prompting public health action, New York City, NY, USA, June–July 2020*

Maximum temporal window applied, d	Specimen collection date range	Detection date†	Radius, km	Observed cases	Relative risk	Recurrence interval, d	SARS-CoV-2 positivity within cluster, %	Median age (range), y	Notes
7	Jun 17–19	Jun 22	0.6	6	4.0	1	2.2	40 (28–58)	Low recurrence interval; epidemiologic linkage of a gathering identified
21	Jul 5–12	Jul 15	0.6	20	3.4	55	8.9	34 (4–87)	Cluster contributed to selection of area for geographically targeted testing, outreach, and education

*SARS-CoV-2, severe acute respiratory syndrome coronavirus 2. †To account for data accrual lags, a 3-d delay was imposed between the end of the SaTScan (https://www.satscan.org) study period and the detection date.

**Figure:**
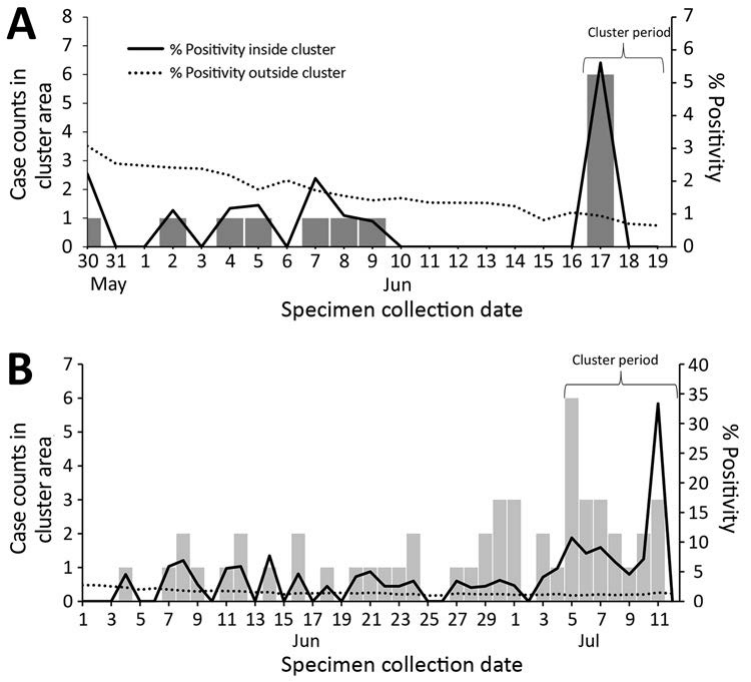
Cluster case counts and severe acute respiratory syndrome coronavirus 2 test percent positivity inside and outside cluster area for selected clusters detected in New York City, NY, USA, 2020. A) Cluster detected on June 22, 2020, in 5 census tracts in which patients reported common attendance at a social gathering; B) cluster detected on July 15, 2020, in 7 census tracts, contributing to the selection of 1 area for targeted testing and outreach.

## Conclusions

COVID-19 community clusters detected by SaTScan prompted localized public education, testing, and community engagement (*15*). In addition, prioritizing interviews of patients in clusters can identify epidemiologic linkages and opportunities for interrupting further transmission, as is done for other reportable diseases (*6*–*8*). Identification of only 1 linkage in this study could be attributable to changing cluster investigation protocols, low patient response rates, or transmission occurring diffusely in small gatherings. Because all patients are referred for contact tracing, DOHMH discontinued reactively interviewing cluster patients for linkages and instead used clusters to proactively target resources.

The first limitation in this study was timeliness. Analyses were based on specimen collection date; however, given delays in testing availability and care seeking, these dates did not necessarily represent recent infections. Timeliness was further limited by delays from specimen collection to laboratory testing and reporting. Clusters dominated by asymptomatic patients or patients with illness onset >14 days before diagnosis may not require intervention because positive PCR results indicate presence of viral RNA but not necessarily viable virus. The second limitation involved the need to geocode for spatial precision. Of unique NYC residents for whom a specimen was collected for SARS-CoV-2 RNA PCR testing during June–July 2020, residential address was not geocodable for ≈3% of residents, so they were excluded. Third, although recurrence interval thresholds can be used to prioritize responding to clusters (*6*), COVID-19 cluster interpretation can be more complex. Other characteristics for prioritizing COVID-19 clusters, besides statistical significance, include percent positivity, relative risk, case count, epidemic curve trajectory, radius, demographics, and persistence. Prioritization can differ by response activity (e.g., establishing new testing sites, conducting outreach) and how quickly resources can be reallocated. Deciding when and where to initiate interventions in response to COVID-19 clusters cannot be fully automated and requires epidemiologic interpretation.

In summary, our COVID-19 early detection system highlighted areas warranting a rapid response. Targeted, place-based approaches for education and outreach efforts and for localized high transmission warnings could better protect persons at high risk for severe illness and death.

Appendix Supplementary methods for detecting COVID-19 clusters at high spatiotemporal resolution, New York City, NY, USA, June–July 2020.
