# Supplementary material for: Detecting COVID-19 Clusters at High Spatiotemporal Resolution, New York City, New York, USA, June–July 2020
Source: Emerg Infect Dis. 2021 May;27(5):1500–4. doi: 10.3201/eid2705.203583 (PMC8084513; doi:10.3201/eid2705.203583)
Supplement: Appendix — Supplementary methods for detecting COVID-19 clusters at high spatiotemporal resolution, New York City, NY, USA, June–July 2020. [file 20-3583-Techapp-s1.pdf]

Article DOI: https://doi.org/10.3201/eid2705.203583

Detecting COVID-19 Clusters at High Spatiotemporal Resolution, New York City, NY, USA, June–July 2020

Appendix

Geocoding

Patient addresses were geocoded daily using version 20A of the NYC Department of City Planning's Geosupport geocoding software, implemented in R through C++ using the Rcpp package (*1*). Addresses that failed to geocode were then cleaned using a string searching algorithm performed against the Department of City Planning's Street Name Dictionary and Property Address Directory. Addresses that failed to geocode after cleaning were then verified using the IBM Infosphere USPS service.

Reference

1. Eddelbuettel D, Francois R. Rcpp: seamless R and C++ integration. J Stat Softw. 2011;40:1–18. https://doi.org/10.18637/jss.v040.i08

Appendix Table. Analysis parameter settings for SARS-CoV-2 test percent positivity cluster detection analyses in New York City, June–July 2020, using the prospective Poisson-based space-time scan statistic.

Parameter	Parameter setting	Notes
Analysis type	Prospective space-time	For timely cluster detection, prospective (rather than retrospective) analyses are used, evaluating only the subset of possible clusters that encompass the last day of the study period. To detect acute, ongoing, localized disease clusters, space-time analyses (rather than purely temporal or purely spatial analyses), are used
Model type	Discrete Poisson	We apply the discrete Poisson-based scan statistic, defining the "population" file as persons tested, to scan for clusters of increased percent positivity. If SARS-CoV-2 percent positivity is high (say, >10%), then the discrete Poisson-based scan statistic is a poor approximation for Bernoulli-type data of persons testing positive and negative. The analysis would produce conservative p-values (i.e., recurrence intervals biased too low). However, for the June–July 2020 period described herein, citywide percent positivity was low, at <4%, so the Poisson model was a very good approximation of the Bernoulli model. Spatial and temporal adjustments for the Bernoulli probability model will be included in a forthcoming SaTScan release and would be preferred in the context of high percent positivity.
Maximum spatial cluster size	50% of the population being tested	The option that imposes the fewest assumptions is to allow the cluster to expand in size to include up to 50% of all persons tested during the study period. Forcing clusters to be smaller than 50%, or restricting in terms of geographic size by setting a maximum circle radius, can be motivated in geographically larger study regions.

Parameter	Parameter setting	Notes
Maximum temporal cluster size	7 d for analysis to support prioritization of case investigations; or 21 d for analysis to support place-based resource allocation	To focus on hotspots emerging during the most recent week; or to focus on areas with more sustained emerging increases.
Minimum temporal cluster size	3 d for analysis to support prioritization of case investigations; or 7 d for analysis to support place-based resource allocation	Clusters of <3-d duration considered less credible for investigation as an emerging hotspot; or clusters of <7 d considered lower priority for resource allocation.
Minimum number of cases	5 cases	Require a minimum number of cases to improve the probability of at least 3 patients within a given cluster being reachable for interview to support identification of a common exposure, or so that resources are not targeted to small numbers of patients.
Temporal trend adjustment	Log-linear with automatically calculated trend	If citywide percent positivity decreasing overall, then wish to detect areas where decreasing slower than citywide average. If citywide percent positivity increasing overall, then wish to detect areas where increasing more than citywide average. A log-linear trend adjustment was suitable for the June–July 2020 period described herein (see percent positivity outside cluster trends in Figure), but when the trend is not log-linear, a different temporal trend adjustment (e.g., log-quadratic, nonparametric) should be used.
Spatial adjustment	Nonparametric, with spatial stratified randomization	The goal during June–July 2020 was to detect areas with relative increases from baseline, even if still lower than average citywide. This method adjusts the expected count separately for each location, removing all purely spatial clusters. The randomization is then stratified by location ID to ensure that each location has the same number of events in the real and random datasets.
Scan for areas with:	High rates	Interested only in increased disease transmission.
Inference	Default p-value method, with maximum number of Monte Carlo replications = 9999	A maximum of 9999 replications increases power compared with 999 replications and is computationally feasible.
Boscoe’s limit of clusters by risk level	None	SaTScan can detect clusters with a relative risk near 1, which are not necessarily useful from a public health perspective. It is possible to restrict the analysis to only detect high rate clusters with a minimum relative risk. We did not use this setting during the June–July 2020 period described herein, but in the context of increasing percent positivity, setting a minimum relative risk (e.g., ≥2.0 or 2.5) limits clusters with large spatial extents.
Secondary cluster reporting criteria (output parameter)	No cluster centers in other clusters	COVID-19 may have multiple active clusters at any moment, so secondary clusters should be reviewed. By reviewing clusters with no cluster centers in other clusters (rather than no, or more geographic overlap), secondary clusters with some overlap can be detected. There is no biologically plausible reason to require secondary clusters to have no geographic overlap.
